# Metacognitions and brooding predict depressive symptoms in a community adolescent sample

**DOI:** 10.1186/s12888-022-03779-5

**Published:** 2022-03-01

**Authors:** Helene Pedersen, Ingrid Grønnæss, Mons Bendixen, Roger Hagen, Leif Edward Ottesen Kennair

**Affiliations:** 1 Department of Psychology, Norwegian University of Science and Technology, Trondheim, Norway; 2 Department of Psychology, University of Oslo, Oslo, Norway; 3 Research institute, Modum Bad, Vikersund, Norway

## Abstract

Depressive symptoms are prevalent in adolescence, and girls have higher levels of depressive symptoms and depressive disorder than boys. Rumination and especially brooding, seem to be a central maintaining factor of depressive symptoms, where metacognitions about rumination play a prominent role in maintaining depressive rumination. There is a sex difference in adults in depressive disorder. The current investigation of a high school / community sample of adolescents aged 16–20 from Norway (*N* = 1198, 62.2% women) found that adolescent women had higher scores than men on all relevant measures: Depressive symptoms, negative and positive metacognitions, pondering, and brooding. A path model for predicting depressive symptoms showed that the major factors for both sexes were negative metacognitions and brooding. The predictors of depressive symptoms were invariant across sex and age groups, suggesting similar underlying mechanisms across these groups. The overall findings suggest that metacognitive therapy may be an efficient intervention for depressive symptoms among adolescents.

## Introduction

Depressive symptoms have a high prevalence in adolescence [8, 11, 27, 29], and research suggest that depressive symptoms in adolescence predict depression in adulthood [1, 57, 66]. Additionally, depression is rated by the World Health Organization [75] as the leading cause of disability among adults worldwide. During early [19] and middle [4, 39] adolescence the incidence of depressive symptoms increases. Additionally, the sex difference in depressive symptoms increases during early to middle adolescence [6, 65], where adolescent women have considerably higher levels of depressive symptoms compared to adolescent men [29, 65]. This gap is particularly prominent after girls’ menarche [12], from 12 to 15 years. However, there is scarce research that has investigated how and why the sex difference in depressive symptoms alters from middle to late adolescence. Using a large sample of high school students, we will address the interaction of gender and age on rumination and the effects of metacognitions on depressive symptoms.

Metacognitive therapy is found to be effective in treating depression [30, 31, 60]. The metacognitive approach to depression builds upon the Self-Regulatory Executive Function model (S-REF [68, 72, 73];). In line with the S-REF model, psychological disorders develop and are maintained due to unhelpful regulatory strategies called the Cognitive Attention Syndrome (CAS; [69, 72]) and metacognitive beliefs about rumination. From a metacognitive perspective, rumination is the primary factor in the development and maintenance of depression [47, 72]. Rumination is a style of thinking that involves repetitive thinking about personal problems [69]. Prospective studies have shown that elevated levels of rumination could lead to depression in children [2] and adolescents [9, 14]. In addition, rumination is found to impact the onset of depressive episodes in children [23] and adults [46, 47]. Further, meta-analyses have shown that there is a robust positive association between rumination and depression [5, 51, 67].

Rumination may be divided into two factors: brooding and pondering [62]. Brooding refers to a passive way of comparing the present situation with more desirable and unattainable standards and is associated with more depressive symptoms [25, 58, 62]. Pondering refers to more purposeful cognitive problem-solving strategies and is associated with fewer depressive symptoms over time [62].

Adolescent and adult women ruminate more than adolescent and adult men [3, 17, 34, 35, 40, 43, 44, 62], including both brooding and pondering [14, 34, 62]. When men and women have high levels of brooding, this is proposed to be an important predictor for elevated levels of depressive symptoms [41, 44]. Concurrently, the sex difference in depressive rumination (i.e., brooding) might account for the difference between men and women in levels of depressive symptoms [35, 44, 45].

As mentioned above, depressive rumination is maintained by metacognitions about rumination [52, 53, 69]. Metacognitions are positive and negative beliefs about cognitive processes and coping strategies [68, 69]. Positive metacognitions related to depressive rumination are beliefs that rumination is an adaptive coping strategy in order to cope with negative thoughts and feelings (e.g., “ruminating about the past helps me to prevent future mistakes and failures”) [53, 68, 69]. Negative metacognitions concern different aspects related to the belief about the uncontrollability of rumination (e.g., “Rumination about my problems is uncontrollable”) [52, 68, 69]. Both positive and negative metacognitions are central components in the maintenance of depression [31, 52, 68], however negative metacognitions may be the best predictor of brooding [55].

Irak [33] and Tosun and Irak [61] found that adolescent women have higher levels of metacognitions compared to adolescent men, whereas others found no sex differences [18, 20, 37, 74]. However, there seems to be elevated levels of metacognitions in women compared to men, and that adolescent and adult women show higher levels of rumination and depressive symptoms compared to adolescent and adult men [14, 29, 34, 35, 43, 44, 62, 65, 75].

### Aims and hypotheses

The current study aims to explore sex differences in depressive symptoms in adolescence, and how depressive symptoms vary across age groups during late adolescent years. Another aim is to explore the associations between metacognitions, rumination, and depressive symptoms as measured with The Beck Depression Inventory (BDI) in adolescence. More specifically our hypotheses were: (H1) Adolescent women would report higher levels of depressive symptoms, metacognitions, and rumination than adolescent men, (H2) Metacognitions would predict rumination, and particularly that negative metacognitions would predict brooding, which again predict elevated depressive symptoms. Hence, the factors predicting depressive symptoms are expected to be equal for adolescent men and women. In addition, we examine the following research questions: (RQ1) To what extent do the predictors of depressive symptoms vary across age groups during late adolescent years (i.e., consistency of the model)? and (RQ2) Is the effect of metacognitions on depressive symptoms direct or is the effect accounted for by the rumination factors?

## Method

### Participants

A convenience sample of high-school students from two Mid-Norwegian counties were asked to complete a web-based questionnaire. A total of 1418 returned their responses. Students aged 21 or older were excluded (*n* = 44), and so were students not identifying as man or woman (*n* = 22). In addition, those who dropped out during responding and who did not fill in the final part of the questionnaire (*n* = 176) were excluded. Finally, we also excluded *n* = 78 following screening procedures for monotonous responding. The final sample eligible for analysis was *n* = 1198 (87.2% of the total sample). This exclusion did not affect the overall level of depressive symptoms (BDI). The excluded group reported somewhat lower levels of symptoms compared to those included (d = 0.24). The total sample consisted of 63.2% women and 36.8% men, with a mean age of 18.2 (*SD* = 1.1) ranging from 16.1 to 20.9.

### Procedure

Data collection took place between February and August 2019. Principals, school nurses, counsellors, and psychology teachers working at high schools in two counties were asked for participation. Half of the high schools agreed to let their students participate. We contacted the principals at the attending schools for distributing information about the study to the students along with a link to the questionnaire. The same information was distributed to the practice students in vocational studies by the county council authorities. The participants received no incentives for responding to the questionnaire. All schools were offered a lecture or a video lecture on mental health in adolescence and preliminary findings of this study for participating. Information regarding the study was reiterated on the welcome page of the questionnaire ensuring confidentiality and that all responses would be subject to anonymization (i.e., any possible personal identification including IP-address were removed) before being subject to any analyses. Participants were informed of their right to discontinue whenever they wanted during responding. Finally, we provided information about mental health care services. Following Norwegian law all participants above 16 years of age provided informed consent. The Regional Committee for Medical and Health Research Ethics for Central Norway approved the study and design (ref. no. 2017/2062). The dataset analysed during the current study is available from the corresponding author on reasonable request. All methods were carried out in accordance with relevant guidelines and regulations.

### Measurements

The measurements below are presented in the same order as they appeared in the questionnaire. In addition to sex, age, and relationship status, the participants reported on their contact with mental health care services for depression (no/yes), and on their antidepressant use (no/yes).

The Beck Depression Inventory (BDI; [10]) was used to assess depressive symptoms. The BDI consists of 21items related to cognitions, behaviour and feelings typically associated with depression. For each item, the respondents rated their level of symptom intensity on four-point scale ranging from 0 to 3, where higher scores indicate more depressive symptoms. The BDI is found to be a valid measure for levels of depressive symptoms in adolescent clinical and non-clinical populations [10]. Internal consistency (Cronbach’s alpha) in this study was excellent (0.93). The following guidelines for BDI cut-off scores were used: Scores below 10 indicate none or minimal depression. Scores between 10 and 18 indicate mild to moderate depression, between 19 and 29 indicate moderate to severe depression, and 30 and higher severe depression (Beck, Steer & Carbin, [10]).

The short version of Ruminative Response Scale (RRS; [62]) was used to assess ruminative brooding and pondering. Also, we removed two items that contained the word “depression”, leaving five brooding items and three pondering items for analyses (see [7]). Instructions read: for the RSS “Consider the last times you were in a situation where you felt sad or had a problem”. For each item the participants gave their responses on a 4-point rating scale, with scores ranging from 1 (*Almost never*) to 4 (*Almost always*). Internal consistency for the five brooding items was good (α = 0.82), but slightly below 0.70 for the three pondering items (α = 0.66). Removal of the item “Write down what you are thinking and analyze it” increased the internal consistency (Spearman-Brown = 0.75). We decided to use the remaining two pondering items. Item scores were averaged to form two scales reflecting brooding and pondering, where higher scores reflect more frequent rumination.

The Positive Beliefs about Rumination Scale (PBRS [53];) was used to measure positive beliefs about rumination (e.g., “Ruminating about the past helps me to prevent future mistakes and failures”). We applied four of the original nine PBRS items. The item selection was done to avoid a possible confounding problem of the word depression [7]. Each item was rated on a 4-point rating scale, ranging from 1 (*disagree*) to 4 (*totally agree*). Internal consistency was good (α = 0.77). Item scores were averaged to form a positive metacognitive belief scale.

The Negative Beliefs about Rumination Scale **(**NBRS; [52]) was used to assess for negative metacognitions (e.g., “Rumination about my problems is uncontrollable” and “People will reject me if I ruminate”). The 13-item scale was used. Each item was rated on a 4-point rating scale, ranging from 1 (*disagree*) to 4 (*totally agree*)*.* Internal consistency was excellent (α = 0.90). Item scores were averaged to form a negative metacognitive belief scale.

### Statistical analyses

**Fig. 1:**
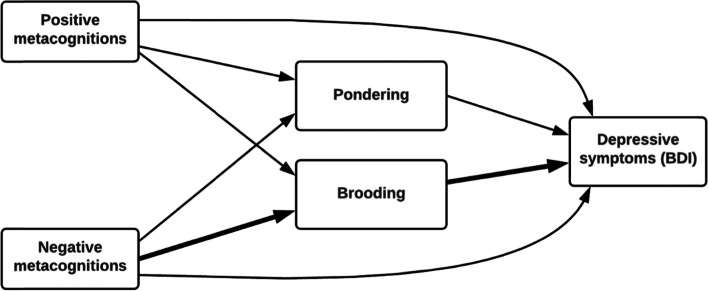
The tested model for men and women and across age groups. Bold lines indicate stronger predicted associations. All associations are predicted to be positive

T-tests were applied for analyses of sex differences along with Hedges’ *g* for effect sizes. Zero-order correlations (Pearson’s *r*) were reported separately for men and women for bivariate associations among predictors and the outcome variable. For predicting depressive symptoms in the outlined model (Fig. 1) we applied Path analysis using Structural Equation Modelling (SEM) on observed variables in Stata/MP 16.1 for Mac (StataCorp, 2019) with robust estimation of standard errors. Path analysis is an extension of the regression model for observed variables and permits a complete test of a specified model with groups and mediators. Stata/MP 16.1 allows for direct tests of moderation effects through tests for group (men vs. women) invariance of parameters and returns Chi-Square values from Ward tests. We also tested possible mediation effects using the MEDSEM module in Stata [38].

## Results

### Gender differences

To test Hypothesis 1 on sex differences in depressive symptoms, metacognitions, and rumination we ran a number of independent samples t-tests (see Table 1 for means and SDs). Women reported moderately more depressive symptoms than men, *t*(1193) = 8.60, *p* < .001, *g* = 0.52. Still, 65.8% of women and 40.5% of men reported BDI scores above the mild to moderate cut-off (scores ≥10). Markedly more women (13.4%) than men (5.2%) scored above the cut-off for severe depression (scores ≥30), which translates to an odds ratio of 2.8. This sex difference was further sustained by more women (11.2%) than men (6.4%) reporting being in contact with mental health care services for depression. Self-reported prevalence of antidepressant use was also higher for women (4.4%) than for men (2.5%). Women also scored higher than men on both positive (PBRS) and negative (NBRS) meta-cognitions. For PBRS the sex effect was marginal but significant, *t*(1193) = 2.09, *p* = .037, *g* = 0.13. The sex effect for NBRS was moderate, *t*(1194) = 6.94, *p* < .001, *g* = 0.42. Women also reported moderately more ruminative behaviors than men: Pondering *t*(1195) = 8.28, *p* < .001, *g* = 0.50; Brooding, *t*(1196) = 11.33, *p* < .001, *g* = 0.68.

**Table 1:** Means, SDs, and Zero-order Correlations (Pearson’s r) among the Variables. Listwise Deletion. Men: *N* = 432, Women: *N* = 728

	Variables	*M*	*SD*	Age	PBRS	NBPS	Pondering	Brooding
Men	Age	18.36	1.16	–				
	PBRS	1.92	0.72	.15**	–			
	NBRS	1.42	0.53	.15**	.32***	–		
	Pondering	1.98	0.89	.17***	.41***	.59***	–	
	Brooding	1.89	0.70	.15***	.37***	.69***	.68***	–
	BDI	10.07	9.85	.12*	.26***	.71***	.50***	.66***
Women	Age	18.05	1.12	–				
	PBRS	2.01	0.70	−.03	–			
	NBRS	1.66	0.61	−.03	.31***	–		
	Pondering	2.40	0.90	−.04	.37***	.45***	–	
	Brooding	2.38	0.76	−.04	.33***	.67***	.56***	–
	BDI	15.54	11.22	−.03	.27***	.76***	.42***	.66***

*Note.* *** *p* < .001, ** *p* < .01, * *p* < .05. *PBRS* Positive Beliefs about Rumination Scale, *NBRS* Negative Beliefs about Rumination Scale, *BDI* Becks Depression Inventory

### Associations

Before testing Hypothesis 2 we examined the associations between depressive symptoms, metacognitions, rumination, and age. Age was positively and significantly associated with both metacognitions, pondering, and brooding for men but not for women. As shown in Table 1, the associations were small. The remaining bivariate associations did not differ between men and women. Positive (PBRS) metacognitions correlated moderately with negative (NBRS) metacognitions. Strong associations were found between the NBRS and brooding, and between brooding and pondering. Negative metacognitions and brooding were also strongly associated with depressive symptoms.

### Predictors of depressive symptoms

**Fig. 2:**
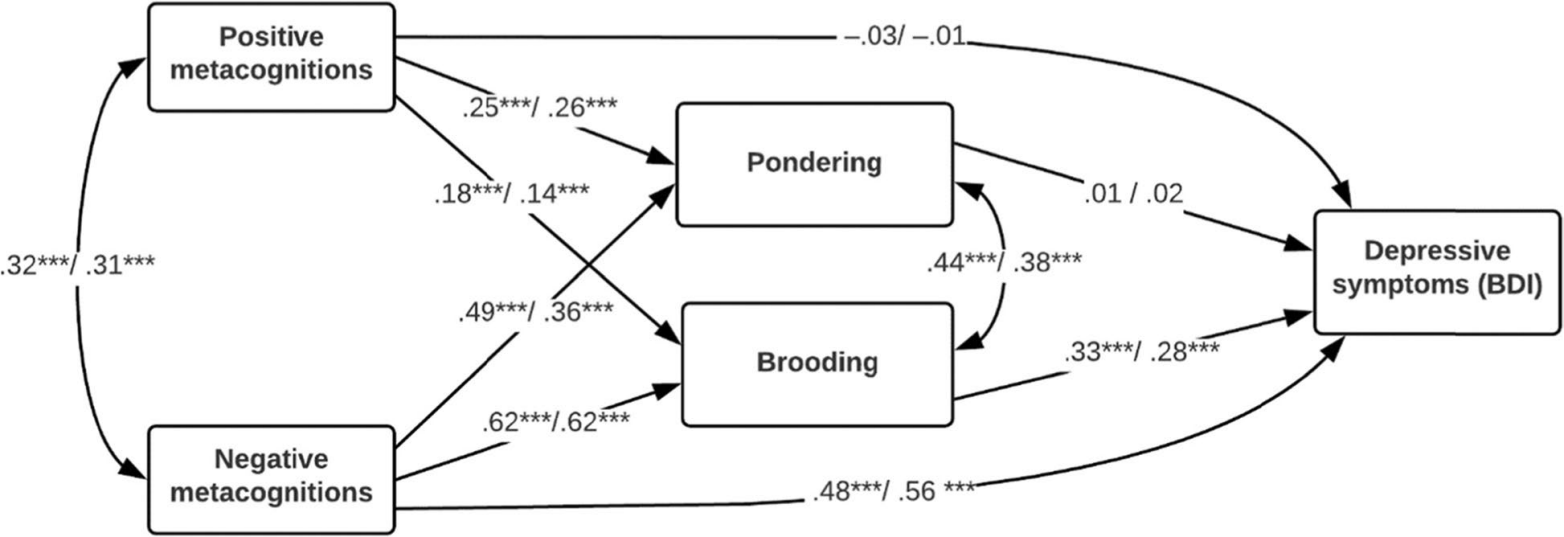
Standardized path coefficients for predictors of depressive symptoms. ***p < .001. Coefficients are presented as men / women

The path model (see Fig. 2) provided strong support for Hypothesis 2. For both men and women, we found that negative metacognitions (NBRS) strongly predicted brooding, and that brooding in turn was the best predictor of depressive symptoms. The two metacognitive and the two rumination scales accounted for a sizable amount of variance in depressive symptoms (BDI) for both sexes (men: 55.1%; women: 61.5%). NBRS alone accounted for 47.9 and 55.5% of the variance in BDI for men and women, respectively (direct and indirect effects). Brooding was primarily predicted by NBRS (38.9 and 38.6% of the explained variance for men and women respectively). In the model, positive metacognitions (PBRS) accounted for only 3.2 and 1.9% to the variance in brooding for men and women, respectively. Separate mediation analyses suggest that the effect of NBRS on BDI was partly accounted for by the effect of brooding (30.0 and 23.6% in men and women, respectively).^1^ Pondering did not significantly mediate the effect of NBRS on BDI.

Tests of parameter invariance suggest that the paths to BDI were not significantly different for men and women. However, the model was not completely similar across sexes. The effect of NBRS on pondering was stronger for men (*χ*^2^ = 9.32, *p* = 0.002). We finally examined to what extent the predictors of depressive symptoms varied across age groups during late adolescent years testing for group invariance in the parameters in the model. When we compared the model for the age cohorts 16 (*n* = 223), 17 (*n* = 324), 18 (*n* = 287), and 19–20 (*n* = 332), none of the parameters were significantly different despite sex differences in BDI tended to decrease monotonically with increasing age from *g* = 0.73 in age group 16 to *g* = 0.36 in age group 19–20.

## Discussion

More women than men are diagnosed with depressive disorder [75]. This difference is thought to arise during adolescence [4, 6, 19, 39, 65]. The major psychological maintenance factor for depression is rumination, especially brooding [47, 72]. A new treatment model for depression also highlights the role of metacognitions, in particular negative metacognitions [69]. The current study therefore considers mechanism invariance across sex and four age groups of adolescents, focusing on the role of metacognitions and rumination on depressive symptoms.

In support of Hypothesis 1, we found that adolescent women reported higher levels of depressive symptoms, stronger positive and negative metacognitions, and higher levels of pondering, and brooding than adolescent men. The overall sex difference in depressive symptoms was moderate, and evident across age groups, but shrunk with increasing age. However, markedly more women than men had scores indicating depressive disorder. Levels of depressive symptoms were high in our sample. This corresponds to the increased levels of depressive symptoms reported by adolescents during the last decades as suggested by recent findings from Norway [8, 13] and other western countries [63, 64]. In line with this, the current study found higher levels of depressive symptoms compared to older non-clinical samples of adolescents [15, 24, 32] However, the sex difference between adolescent men and women scoring above clinical cut-offs was as expected.

There could be different explanations to such sex differences related to levels of depressive symptoms. One could be that women experience more stressors than men in adolescence, and therefore become more depressed [28]. Research has shown that women report more interpersonal stressors (including in relation to peers and romantic partners), whilst boys experience more achievement and self-relevant stressors [28]. Another explanation could be related to how the sexes cope with emotional distress. Men seem to use more alcohol or other coping strategies related to self-regulation to alleviate distress, while women express distress more in the form of depressive symptoms [41, 42].

The main predictor of depressive symptoms was negative metacognitions. The effect of this predictor was strong and only partly mediated by brooding. This is supportive of Hypothesis 2 and RQ1. It is not surprising that negative metacognitions predict depressive symptoms; this is found in other studies, too [16, 59]. According to the metacognitive model of depression, rumination is maintained by metacognitions. If individuals experience that they have problems in exercising appropriate control over negative affective experiences [69], this is likely to result in maladaptive coping strategies, including more rumination and therefore heightened levels of depressive symptoms.

The path model was consistent across sex and across age groups, suggesting that the predictors of depressive symptoms were similar for adolescent men and women, and similar across age. The effect of age on men’s but not women’s BDI scores may suggest that depressive symptoms and the maintaining mechanisms identified in the current study come online during puberty, and that later maturation of emotional regulation in adolescent men [22] results in their increasing scores across the current age groups.

### Limitations and future research

Despite the large sample size, the representativeness of the sample is unknown, albeit both urban and rural schools, and both academic and vocational studies were sampled. Studies from other countries are warranted, as generalizability of these findings may be culture dependent. In addition, the current study has low response rate which is common for studies in this age group using similar methodology [36, 56]. Despite this, the overall level of depressive symptoms above BDI cut-offs and the sex differences in the different indicators of depression is favourable of the representativeness of the sample. However, while lack of representativeness may be a problem for levels of symptoms and group effects, associations and mechanisms under study need not be affected by this [21]. Still, mediation analyses from cross-sectional studies need to be interpreted with caution because the temporal sequence of the process under study is not known, and cross-sectional data may produce very different results than longitudinal data [50].

Future research should explore lower age groups to identify when the sex difference in depressive symptoms appears and whether the metacognitive model holds for even younger age groups. We further recommend that future research reproduces the findings in a clinical population.

## Conclusion and clinical implications

This first investigation of the invariance of the effect of rumination and metacognitions on depressive symptoms across sex and age group, support the metacognitive model of depressive symptom maintenance. Adolescent women had higher levels of depressive symptoms, metacognitions, and brooding than adolescent men. There was support for a metacognitive model of depression in a non-clinical adolescent community sample aged 16 to 20. In line with prior research, brooding and negative metacognitions were central components in the development and maintenance of depressive symptoms [54, 69, 72]. Further, there was no effect of age. The model was invariant across groups of adolescents aged 16 to 20. Brooding and negative metacognitions are important targets for clinical interventions aimed at reducing depressive symptoms among adolescents.

The results could have clinical implications. Recent studies have shown support for the use of metacognitive therapy (MCT) when treating depression in adults [26, 30, 31, 48, 49, 60, 70, 71]. Since rumination also seems to be closely related to depressive symptoms in adolescence, the current results indicate that MCT might be a beneficial choice of therapy when treating depression in these age groups.

## Data Availability

The dataset analysed during the current study is available from the corresponding author on reasonable request.

## Footnotes

1 We also tested the opposite pattern; to what extent did negative metacognitions (NBRS) mediate the effect of brooding on depressive symptoms (BDI). The results suggest that the mediation effect of NBRS (43.6% for men and 55.3% for men and women) was stronger than that of brooding.

## References

[1] Aalto-Setälä T, Marttunen M, Tuulio-Henriksson A, Poikolainen K, Lönnqvist J (2002). Depressive symptoms in adolescence as predictors of early adulthood depressive disorders and maladjustment. Am J Psychiatry.

[2] Abela JR, Brozina K, Haigh EP (2002). An examination of the response styles theory of depression in third-and seventh-grade children: A short-term longitudinal study. J Abnorm Child Psychol.

[3] Abela JR, Parkinson C, Stolow D, Starrs C (2009). A test of the integration of the hopelessness and response styles theories of depression in middle adolescence. J Clin Child Adolesc Psychol.

[4] Abela JRZ, Hankin BL (2011). Rumination as a Vulnerability Factor to Depression During the Transition From Early to Middle Adolescence: A Multiwave Longitudinal Study. J Abnorm Psychol.

[5] Aldao A, Nolen-Hoeksema S, Schweizer S (2010). Emotion-regulation strategies across psychopathology: A meta-analytic review. Clin Psychol Rev.

[6] Angold A, Erkanli A, Silberg J, Eaves L, Costello EJ (2002). Depression scale scores in 8–17-year-olds: effects of age and gender. J Child Psychol Psychiatry.

[7] Armey MF, Fresco DM, Moore MT, Mennin DS, Turk CL, Heimberg RG, Alloy LB (2009). Brooding and pondering: Isolating the active ingredients of depressive rumination with exploratory factor analysis and structural equation modeling. Assessment.

[8] Bakken A. Ungdata 2018 Nasjonale resultater (NOVA rapport 8/2018). Retrieved November 21, 2020, from http://www.hioa.no/Om-OsloMet/Senter-for-velferds-og-arbeidslivsforskning/NOVA/Publikasjonar/Rapporter/2018/Ungdata-2018.-Nasjonale-resultater.

[9] Bastin M, Mezulis AH, Ahles J, Raes F, Bijttebier P (2015). Moderating effects of brooding and co-rumination on the relationship between stress and depressive symptoms in early adolescence: A multi-wave study. J Abnorm Child Psychol.

[10] Beck AT, Steer RA, Carbin MG (1988). Psychometric properties of the Beck Depression Inventory: twenty-five years of evaluation. Clin Psychol Rev.

[11] Bhatia SK, Bhatia SC (2007). Childhood and adolescent depression (Disease/Disorder overview). Am Fam Phys.

[12] Birmaher B, Ryan ND, Williamson DE, Brent DA, Kaufman J, Dahl RE, Nelson B (1996). Childhood and adolescent depression: a review of the past 10 years. Part I. J Am Acad Child Adolesc Psychiatr.

[13] Blaauw BA, Dyb G, Hagen K, Holmen TL, Linde M, Wentzel-Larsen T, Zwart JA (2015). The relationship of anxiety, depression and behavioral problems with recurrent headache in late adolescence–a Young-HUNT follow-up study. J Headache Pain.

[14] Burwell RA, Shirk SR (2007). Subtypes of rumination in adolescence: Associations between brooding, reflection, depressive symptoms, and coping. J Clin Child Adolesc Psychol.

[15] Canals J, Bladé J, Carbajo G, Domènech-Labería E (2001). The Beck Depression Inventory: Psychometric characteristics and usefulness in nonclinical adolescents. Eur J Psychol Assess.

[16] Cano-López JB, Salguero JM, García-Sancho E, Ramos-Cejudo J (2021). Testing the Metacognitive Model of Rumination and Depression in Non-clinical Population: New Data about PBRS and NBRS Scales. J Psychopathol Behav Assess.

[17] Carnevali L, Thayer JF, Brosschot JF, Ottaviani C (2018). Heart rate variability mediates the link between rumination and depressive symptoms: A longitudinal study. Int J Psychophysiol.

[18] Cartwright-Hatton S, Mather A, Illingworth V, Brocki J, Harrington R, Wells A (2004). Development and preliminary validation of the Meta-cognitions. Questionnaire—Adolescent Version. J Anxiety Disord.

[19] Costello EJ, Mustillo S, Erkanli A, Keeler G, Angold A (2003). Prevalence and development of psychiatric disorders in childhood and adolescence. Arch Gen Psychiatry.

[20] Crye J, Laskey B, Cartwright-Hatton S (2010). Non-clinical obsessions in a young adolescent population: Frequency and association with metacognitive variables. Psychol Psychother.

[21] Dey EL (1997). Working with low survey response rates: The efficacy of weighting adjustments. Res High Educ.

[22] Frere PB, Vetter NC, Artiges E, Filippi I, Miranda R, Vulser H, Lemaître H (2020). Sex effects on structural maturation of the limbic system and outcomes on emotional regulation during adolescence. NeuroImage.

[23] Gibb BE, Grassia M, Stone LB, Uhrlass DJ, McGeary JE (2012). Brooding rumination and risk for depressive disorders in children of depressed mothers. J Abnorm Child Psychol.

[24] Gorenstein C, Andrade L, Zanolo E, Artes R (2005). Expression of depressive. symptoms in a nonclinical Brazilian adolescent sample. The. Can J Psychiatry.

[25] Grierson AB, Hickie IB, Naismith SL, Scott J (2016). The role of rumination in illness trajectories in youth: linking trans-diagnostic processes with clinical staging models. Psychol Med.

[26] Hagen R, Hjemdal O, Solem S, Kennair LEO, Nordahl HM, Fisher P, Wells A (2017). Metacognitive Therapy for Depression in Adults: A Waiting List Randomized Controlled Trial with Six Months Follow-Up. Front Psychol.

[27] Hankin BL (2006). Adolescent depression: Description, causes, and interventions. Epilepsy Behav.

[28] Hankin BL, Mermelstein R, Roesch L (2007). Sex Differences in Adolescent Depression: Stress Exposure and Reactivity Models. Child Dev.

[29] Hankin BL, Young JF, Abela JR, Smolen A, Jenness JL, Gulley LD, Oppenheimer CW (2015). Depression from childhood into late adolescence: influence of gender, development, genetic susceptibility, and peer stress. J Abnorm Psychol.

[30] Hjemdal O, Hagen R, Solem S, Nordahl H, Kennair LEO, Ryum T, Wells A (2016). Metacognitive Therapy in Major Depression: An Open Trial of Comorbid Cases. Cogn Behav Pract.

[31] Hjemdal O, Solem S, Hagen R, Kennair LEO, Nordahl HM, Wells A (2019). A randomized controlled trial of metacognitive therapy for depression: Analysis of 1-year follow-up. Front Psychol.

[32] Ignjatović-Ristić D, Hinić D, Jović J (2012). Evaluation of the Beck Depression Inventory in a nonclinical student sample. West Indian Med J.

[33] Irak PM (2012). Standardization of Turkish form of metacognition questionnaire for children and adolescents: the relationships with anxiety and obsessive-compulsive symptoms. Turk Psikiyatri Dergisi.

[34] Johnson DP, Whisman MA (2013). Gender differences in rumination: A meta-analysis. Pers Individ Differ.

[35] Jose PE, Brown I (2008). When does the gender difference in rumination begin? Gender and age differences in the use of rumination by adolescents. J Youth Adolesc.

[36] Kessler RC, Avenevoli S, Costello EJ, Green JG, Gruber MJ, Heeringa S, Zaslavsky AM (2009). National comorbidity survey replication adolescent supplement (NCS-A): II. Overview and design. J Am Acad Child Adolesc Psychiatry.

[37] Matthews L, Reynolds S, Derisley J (2007). Examining cognitive models of obsessive compulsive disorder in adolescents. Behav Cogn Psychother.

[38] Mehmetoglu, M. (2017). MEDSEM: Stata module to perform mediation analysis using structural equation modelling (Version S458300): Boston College Department of Economics. Retrieved April 20, 2019, from https://ideas.repec.org/c/boc/bocode/s458300.html.

[39] Newman DL, Moffitt TE, Caspi A, Magdol L, Silva PA, Stanton WR (1996). Psychiatric disorder in a birth cohort of young adults: prevalence, comorbidity, clinical significance, and new case incidence from ages 11 to 21. J Consult Clin Psychol.

[40] Nolen-Hoeksema S (2000). The role of rumination in depressive disorders and mixed anxiety/depressive symptoms. J Abnorm Psychol.

[41] Nolen-Hoeksema S (2012). Emotion regulation and psychopathology: The role of gender Ann Rev. Clin Psychol.

[42] Nolen-Hoeksema S, Corte C. Gender and self-regulation. In: Baumeister RF, Vohs KD, editors. Handbook of self-regulation: Research, theory, and applications. The Guilford Press; 2004. p. 411–21.

[43] Nolen-Hoeksema S, Jackson B (2001). Mediators of the gender difference in rumination. Psychol Women Q.

[44] Nolen-Hoeksema S, Parker LE, Larson J (1994). Ruminative coping with depressed mood following loss. J Pers Soc Psychol.

[45] Nolen-Hoeksema S, Rusting C, Kahneman D, Diener E, Schwarz N (1999). Gender differences in well-being. Foundations of hedonic psychology: Scientific perspectives on enjoyment and suffering.

[46] Nolen-Hoeksema S, Stice E, Wade E, Bohon C (2007). Reciprocal relations between rumination and bulimic, substance abuse, and depressive symptoms in female adolescents. J Abnorm Psychol.

[47] Nolen-Hoeksema S, Wisco BE, Lyubomirsky S (2008). Rethinking Rumination. Perspect Psychol Sci.

[48] Normann N, Morina N (2018). The efficacy of metacognitive therapy: a systematic review and meta-analysis. Front Psychol.

[49] Normann N, van Emmerik AA, Morina N (2014). The efficacy of metacognitive therapy for anxiety and depression: A meta-analytic review. Depression Anxiety.

[50] O’Laughlin KD, Martin MJ, Ferrer E (2018). Cross-sectional analysis of longitudinal mediation processes. Multivar Behav Res.

[51] Olatunji BO, Naragon-Gainey K, Wolitzky-Taylor KB (2013). Specificity of rumination in anxiety and depression: A multimodal meta-analysis. Clin Psychol Sci Pract.

[52] Papageorgiou C, Wells A (2001). Metacognitive beliefs about rumination in recurrent major depression. Cogn Behav Pract.

[53] Papageorgiou C, Wells A (2001). Positive beliefs about depressive rumination: Development and preliminary validation of a self-report scale. Behav Ther.

[54] Papageorgiou C, Wells A (2003). An empirical test of a clinical metacognitive model of rumination and depression. Cogn Ther Res.

[55] Papageorgiou C, Wells A (2009). A Prospective Test of the Clinical Metacognitive Model of Rumination and Depression. International Journal of Cognitive. Therapy.

[56] Patel NC, Delbello MP, Bryan HS, Adler CM, Kowatch RA, Stanford K, Strakowski SM (2006). Open-label lithium for the treatment of adolescents with bipolar depression. J Am Acad Child Adolesc Psychiatry.

[57] Pine DS, Cohen E, Cohen P, Brook J (1999). Adolescent depressive symptoms as predictors of adult depression: moodiness or mood disorder?. Am J Psychiatry.

[58] Satyshur MD, Layden EA, Gowins JR, Buchanan A, Gollan JK (2018). Functional connectivity of reflective and brooding rumination in depressed and healthy women. Cognitive, Affective, & Behavioral. Neuroscience.

[59] Solem S, Hagen R, Hoksnes JJ, Hjemdal O (2016). The metacognitive model of depression: an empirical test in a large Norwegian sample. Psychiatry Res.

[60] Solem S, Kennair LEO, Hagen R, Havnen A, Nordahl HM, Wells A, Hjemdal O (2019). Metacognitive Therapy for Depression: A 3-Year Follow-Up Study. Assessing Recovery, Relapse, Work Force Participation, and Quality of Life. Front Psychol.

[61] Tosun A, Irak M. Adaptation, Validity, and Reliability of the Metacognition Questionnaire-30 for the Turkish Population, and its Relationship to Anxiety and Obsessive-Compulsive Symptoms. Turk Psikiyatri Dergisi. 2008;19(1) 18330745

[62] Treynor W, Gonzalez R, Nolen-Hoeksema S (2003). Rumination reconsidered: A psychometric analysis. Cogn Ther Res.

[63] Twenge JM. Time period and birth cohort differences in depressive symptoms in the US, 1982–2013. Social Indicators Research, 121(2), 437–454. doi. 2015; 10.1007/s11205-014-0647-1.

[64] Twenge JM, Joiner TE, Rogers ML, Martin GN (2018). Increases in depressive symptoms, suicide-related outcomes, and suicide rates among US adolescents after 2010 and links to increased new media screen time. Clinical. Psychol Sci.

[65] Twenge JM, Nolen-Hoeksema S (2002). Age, gender, race, socioeconomic status, and birth cohort difference on the children’s depression inventory: A meta-analysis. J Abnorm Psychol.

[66] Visted E, Sørensen L, Vøllestad J, Osnes B, Svendsen JL, Jentschke S, Schanche E (2019). The Association between Juvenile Onset of Depression and Emotion Regulation Difficulties. Front Psychol.

[67] Visted EV, Vøllestad JJ, Nielsen MM, Schanche EE (2018). Emotion Regulation in Current and Remitted Depression: A Systematic Review and Meta-Analysis. Front Psychol.

[68] Wells A (2000). Emotional disorders and metacognition: Innovative cognitive therapy.

[69] Wells A (2009). Metacognitive therapy for anxiety and depression.

[70] Wells A, Fisher P, Myers S, Wheatley J, Patel T, Brewin CR (2009). Metacognitive therapy in recurrent and persistent depression: A multiple-baseline study of a new treatment. Cogn Ther Res.

[71] Wells A, Fisher P, Myers S, Wheatley J, Patel T, Brewin CR (2012). Metacognitive therapy in treatment-resistant depression: A platform trial. Behav Res Ther.

[72] Wells A, Matthews G (1994). Attention and emotion: A clinical perspective.

[73] Wells A, Matthews G (1996). Modelling cognition in emotional disorder: The S-REF. model. Behav Res Ther.

[74] Wilson C, Budd B, Chernin R, King H, Leddy A, Maclennan F, Mallandain I (2011). The role of meta-cognition and parenting in adolescent worry. J Anxiety Disord.

[75] World Health Organization (WHO) (2018). Depression. Available at: www.who.int/en/newsroom/fact-sheets/detail/depression (accessed 24 Oct 2019).

